# Postmortem diagnostics

**DOI:** 10.1007/s12024-025-01073-w

**Published:** 2025-08-28

**Authors:** Wouter Van Den Bogaert

**Affiliations:** 1 Forensic Biomedical Sciences, Department of Imaging and Pathology, KU Leuven, Leuven, Belgium; 2 Department of Forensic Medicine, University Hospitals Leuven, Herestraat 49, B-3000, Leuven, Belgium

**Keywords:** Forensic pathology, Postmortem diagnostics, Research autopsy, Diagnostic autopsy

 Foster recently described the “autopsy” enigma [1]. At the same time, the term is being used with increasing flexibility and is often misapplied. We argue for a more structured terminology to improve clarity and consistency in postmortem practice.

The word autopsy derives from the Greek for “to see for oneself” and traditionally refers to the internal examination of a deceased person by a pathologist. However, in both clinical use and scientific literature, its meaning has broadened considerably. New technologies and a deeper understanding of disease mechanisms have made the investigation of causes of death more complex. Today, it often extends beyond classical dissection and histology to include various additional procedures, such as postmortem microbiological, biochemical and toxicological analyses, and molecular autopsy (postmortem genetics). The postmortem examination is no longer the exclusive domain of the pathologist but often requires a multidisciplinary team of experts [2]. The word autopsy is now even applied to investigations unrelated to internal examination, such as “verbal autopsies” (structured interviews with relatives) or “virtual autopsies” (postmortem imaging) [3, 4]. This semantic stretch leads to confusion, as stakeholders may use or interpret the term differently.

To better reflect this complex reality, we advocate for the term Postmortem Diagnostics (PMD) as an overarching concept. PMD encompasses *all postmortem investigations conducted to determine the cause and manner of death*,* and to assess the presence and extent of diseases and injuries*.

It is important to distinguish clearly between autopsy practice within PMD and postmortem procedures without diagnostic intent. “Research autopsies”^,^ defined as postmortem medical procedures performed to collect tissue for basic and translational research, do not fall under the definition of PMD and require a distinct legal and ethical framework [5]. Precisely for this reason, the term “diagnostic autopsy” should be introduced as a counterpart, referring to macroscopic and histopathological examinations (as the cornerstone) within PMD. Historically, a binary distinction was made between medico-legal (forensic) autopsies and clinical-scientific (academic) autopsies, based on the legal or hospital context. While still useful for delineating roles and responsibilities, this division becomes less relevant for diagnostic autopsies where clinical and forensic interests converge, such as sudden deaths in the young or novel infectious diseases (as during the COVID-19 pandemic).

As postmortem investigation evolves, so too must the terminology. Clarifying terms such as diagnostic autopsy within the broader framework of postmortem diagnostics will reduce ambiguity in practice and scientific communication.
